# Polarization-Insensitive Transmissive Metasurfaces Using Pancharatnam–Berry and Resonant Phases in Microwave Band

**DOI:** 10.3390/s23239413

**Published:** 2023-11-26

**Authors:** Ling Wang, Yang Yang, Feng Gao, Shuhua Teng, Jinggui Zhang, Li Deng, Weijun Hong, Zhuofang Li

**Affiliations:** 1 School of Electronic Information, Hunan First Normal University, Changsha 410205, China; wangling@hnfnu.edu.cn (L.W.);; 2 Key Laboratory of Hunan Province for 3D Scene Visualization and Intelligence Education, Hunan First Normal University, Changsha 410205, China; 3 School of Electrical and Data Engineering, Tech Lab, University of Technology Sydney, Sydney, NSW 2019, Australia; 4 School of Information and Communication Engineering, Beijing University of Posts and Telecommunications, Beijing 100876, China; dengl@bupt.edu.cn (L.D.);; 5 China Academy of Information and Communications Technology, Beijing 100191, China

**Keywords:** metasurface, polarization insensitive, Pancharatnam–Berry phase, resonant phase, microwave

## Abstract

Most of the existing metasurfaces are effective for the incident wave with the specific circularly polarized (CP) or linearly polarized (LP) state, that is the polarization-sensitive metasurface. This drawback dramatically hinders the practical use of the metasurface. Herein, this paper presents a strategy of polarization-insensitive transmissive microwave metasurfaces to manipulate the incident wave with arbitrary CP and LP states. The metasurface consists of polarization-insensitive unit cells. For the left circularly polarized (LCP) and right circularly polarized (RCP) incident waves, the same abrupt phase covering 0° to 360° can be realized by combining the Pancharatnam–Berry (PB) and resonant phases. As the arbitrary LP wave can decompose into the LCP and RCP waves, metasurfaces consisting of designed unit cells are valid for any polarization states. The polarization-insensitive transmissive microwave metalens and orbital angular momentum multiplexing metasurface working at 23 GHz are devised for verification. Simulation and measurement results verify the availability of the approach. The proposed method is suitable for designing microwave-transmissive metasurfaces capable of polarization insensitivity.

## 1. Introduction

Metasurfaces have attracted significant attention due to their ability to fully control the electromagnetic wave’s phase, magnitude, and polarization [1]. The metasurface is composed of periodic or quasi-periodic unit cells. The required function is realized based on the specific abrupt phase distribution introduced by unit cells. Generally, there are three categories of abrupt phases: the propagation phase, the resonant phase, and the geometric phase, which is also called the Pancharatnam–Berry (PB) phase [2]. The propagation phase based on the phase accumulation is mainly used for the all-dielectric unit cell [3]. The resonant phase is introduced by the unit cell’s resonance [4,5]. The PB phase is only related to the rotation angle of the unit cell and is effective for the circularly polarized (CP) wave [6,7,8].

Much research on the metasurface has been made and various electromagnetic devices are realized based on the metasurface [9], mainly including the metalens [10,11,12,13,14], orbital angular momentum (OAM) generator and multiplexer [15,16,17,18,19], hologram [20], absorber [21], and beam splitter [22]. However, most of the existing metasurfaces are effective for a particular CP [23,24,25,26,27] or linearly polarized (LP) [28,29,30,31,32] incident wave, that is the polarization-sensitive metasurface. This drawback dramatically hinders the practical use of the metasurface. Recently, the polarization-insensitive metasurface has been widely investigated and the research is focused on the optical metasurface. Most of the optical metasurface consists of all-dielectric nanopillars with cylindrical structures. For the incident light wave with any polarization state, the same propagation phase can be obtained. Therefore, the polarization insensitivity of metasurfaces can be achieved [33,34,35]. A few research studies have implemented the polarization-insensitive metasurface combining the PB phase and the propagation phase [36,37]. However, as the propagation phase is used, the profile of these all-dielectric metasurfaces is high, making it difficult to integrate. Further, for the microwave band, obtaining the abrupt phase based on the resonant phase is more effective. Therefore, the polarization-insensitive metasurface working in the microwave band deserves further study.

This paper presents a strategy for polarization-insensitive transmissive microwave metasurfaces. For verification, microwave metalens and OAM multiplexing metasurface with polarization insensitivity are proposed. The abrupt phase profile of the metasurface is formed with the PB and resonant phases simultaneously. The unit cell consists of metal–insulator–metal layers. For the left circularly polarized (LCP) and right circularly polarized (RCP) incident waves, the same PB and resonant phases can be acquired by limiting the orientation and designing specific structures, respectively. As the arbitrary LP wave can be decomposed into the LCP and RCP waves, metasurfaces composed of the proposed unit cells are valid for arbitrary polarization states. The corresponding unit cell parameter with the required resonant phase can be easily calculated by the fitting curve method without redesigning the unit cell, which greatly simplifies the design process. Simulation and measurement results verify the availability of the approach. The proposed method can guide the design of transmissive microwave metasurfaces with polarization insensitivity.

## 2. Theoretical Basis

According to the PB phase principle [6,7,8], the transfer function **T** of the unit cell can be expressed as Equation (1):

(1) $$T=R\left(\alpha \right)J\left(\phi \right)R^{-1}\left(\alpha \right)=\left[\begin{array}{cc} \cos\left(\frac{\phi }{2}\right)-i\cos\left(2\alpha \right)\sin\left(\frac{\phi }{2}\right) & -i\sin\left(2\alpha \right)\sin\left(\frac{\phi }{2}\right)\\ -i\sin\left(2\alpha \right)\sin\left(\frac{\phi }{2}\right) & \cos\left(\frac{\phi }{2}\right)-i\cos\left(2\alpha \right)\sin\left(\frac{\phi }{2}\right) \end{array}\right]$$

where **R**(*α*) is the coordinate rotation matrix and *α* is the local orientation of the axis. **J**(*ϕ*) is the Jones matrix and *ϕ* is the phase delay. Since the arbitrary LP wave can be divided into the LCP and RCP waves, its electric field vector **E**_in_ can be expressed as Equation (2):

(2) $$E_{\mathrm{in}}=\langle E_{\mathrm{in}}|L\rangle +\langle E_{\mathrm{in}}|R\rangle$$

where $|L\rangle$ and $|R\rangle$ denote the LCP and RCP components of **E**_in_, respectively. When the LP wave incidents onto the unit cell, the electric field vector **E**_t_ of the transmitted wave can be expressed as Equation (3):

(3) $$E_{t}=TE_{\mathrm{in}}=\cos\left(\frac{\phi }{2}\right)E_{\mathrm{in}}-i\sin\left(\frac{\phi }{2}\right)\left[\langle E_{\mathrm{in}}|R\rangle e^{-i2\alpha |L\rangle }+\langle E_{\mathrm{in}}|L\rangle e^{i2\alpha |R\rangle }\right]$$

According to Equation (3), for the $\langle E_{\mathrm{in}}|R\rangle$ or $\langle E_{\mathrm{in}}|L\rangle$ incident wave, the cross-polarized component $e^{-i2\alpha |L\rangle }$ or $e^{i2\alpha |R\rangle }$ of the transmitted wave with the additional phase −2*α* or 2*α* will be generated, that is the PB phase. It can be seen that the PB phase is dependent on the polarization state of the incident CP wave. To realize the polarization-insensitive unit cell, *α* is limited to 0° and 90° and the values of $e^{-i2\alpha }$ and $e^{i2\alpha }$ are equal. Therefore, the same 180° PB phase can be obtained under LCP and RCP waves. However, to design the polarization-insensitive metasurface, the abrupt phase introduced by the unit cell should cover 0° to 360°. Therefore, the resonant phase is applied at the same time. The same resonant phase covering the change from 0° to 180° can be obtained by varying parameters of the appropriately designed unit cell. To simplify the design, the fitting relation between the resonant phase and the parameter is attained by the polynomial fitting curve method.

For verification, the metalens and OAM multiplexing metasurface are taken as examples. The abrupt phase profile of the metalens can be expressed as Equation (4):

(4) $$\Phi_{\mathrm{lens}}\left(x,y\right)=\frac{2\pi f}{C}\left[\left(\sqrt{x^{2}+y^{2}+F^{2}}\right)-F\right]$$

where *f* is the frequency of the incident wave, that is the working frequency of the metalens, *C* is the propagation velocity, (*x*, *y*) denotes the arbitrary position on the metalens, and *F* means the required focal length. The abrupt phase profile of the angle-multiplexed metasurface for OAM multiplexing is expressed as Equation (5) [38]:

(5) $$\Phi_{\mathrm{OAM}}\left(x,y\right)=\mathrm{angle}\left(\sum_{m=1}^{M}\exp\left(j\left(l_{m}\arctan\frac{y}{x}+\frac{2\pi f}{C}\sin\left(\theta_{m}\right)\left(x\cos\left(\varphi_{m}\right)+y\sin\left(\varphi_{m}\right)\right)\right)\right)\right)$$

where angle (·) is used to solve the phase angle of the expression in the bracket, *l* is the topological charge of the generated OAM beam, (*θ*, *φ*) represents the azimuth and elevation angles of the oblique incident wave, and *m* indicates the channel number. It is worth noting that the OAM beam has a doughnut intensity profile and helical phase front, and the phase front changes *l*·2π in the direction of rotation. The value of *l* theoretically is unlimited and the OAM beams with different *l* are orthogonal (The topological charge of the plane wave is 0). The topological charge of the generated OAM beam can be calculated by the topological charge purity [39]:

(6) $$\sigma^{2}=\frac{1}{N}\left[{\left(\frac{\phi_{1}-\phi_{N}}{\psi_{1}-\psi_{N}}-l_{s}\right)}^{2}+\sum_{n=2}^{N}{\left(\frac{\phi_{n}-\phi_{n-1}}{\psi_{n}-\psi_{n-1}}-l_{s}\right)}^{2}\right]$$

where *σ*^2^ is the variance with the standard topological charge *l*_s_. For the far-field phase pattern of the OAM beam in the spherical coordinate system, the phase is sampled with the elevation angle *θ*_s_ and the azimuth angle *φ*_s_ changing from 0 to 2π linearly. *ϕ_n_* (*n* = 1, 2,…, *N*) is the sampling phase of the OAM beam, while *ψ_n_* changes from 0 to 2π linearly with the step of 360°/N. By dividing the calculated *σ*^2^ by the maximum value, the normalized *σ*^2^ can be obtained. The higher the topological charge purity, the smaller the normalized *σ*^2^, and the closer the topological charge of the generated OAM beam to *l*_s_.

The design process of the metalens and OAM metasurface: First, the metasurface is set to be composed of *K* × *K* unit cells with a specific period *P*, and (*x*, *y*) is set to the coordinates (*x_i_*, *y_i_*) (*i* = 1, 2, …, *K*) at the center position of unit cells. Second, for the unit cell at (*x_i_*, *y_i_*), the required abrupt phase $\Phi \left(x_{i},y_{i}\right)$ is calculated based on Equations (4) and (5). Next, if $\Phi \left(x_{i},y_{i}\right)$ is less than 180°, the abrupt phase is directly introduced by the resonant phase with *α* = 0°. Otherwise, the abrupt phase is introduced by combining the 180° PB phase with *α* = 90° and $\Phi \left(x_{i},y_{i}\right)$ − 180° resonant phase. At last, the *K* × *K* unit cells with the required abrupt phase are filled into the corresponding positions of the metasurface.

## 3. Numerical Demonstration

### 3.1. Unit Cell

**Figure 1:**
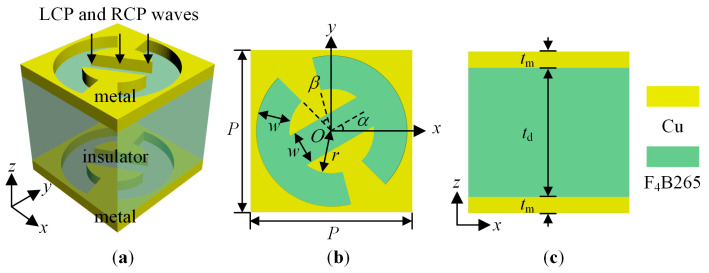
The schematic diagram of the proposed transmissive unit cell. (**a**) Three-dimensional view. (**b**) Top view. (**c**) Side view. *P* = 3 mm, *w* = 0.62 mm, *r* = 0.78 mm, *α* = 0° or 90°, *β* varies between 14° and 139°, *t*_m_ = 0.035 mm, and *t*_d_ = 2.93 mm. The green and yellow colors represent F_4_B265 and Cu respectively.

To verify, a transmissive metallic unit cell working at 23 GHz is proposed. The schematic diagram of the unit cell is shown in Figure 1. The unit cell consists of metal–insulator–metal layers. The upper and lower resonators are the same split-ring resonator (SRR) metal slots. The SRR is one of the most common and practical resonant structures. The metal is lossy copper (Cu) and the electrical conductivity equals 5.8 × 10^7^ S/m. The insulator is F_4_B265 [40] and the relative dielectric constant and loss tangent are set to 2.65 and 0.001, respectively. In Figure 1 the green and yellow colors represent F_4_B265 and Cu respectively. The thicknesses of the metal and insulator are *t*_m_ = 0.035 mm and *t*_d_ = 2.93 mm, respectively. Note that the metal layers are rescaled (thicker) in the schematic for better visualization of the structure [Figure 1a,c]. The period *P* of the unit cell is 3 mm, the width *w* of the slot is 0.62 mm, and *r* = 0.78 mm. According to the design principle, the rotation angle *α* is set to 0° or 90°. To make the resonant phase covering 0° to 180°, the open angle *β* of the SRR slot varies between 14° and 139°. The unit cell is simulated by the CST STUDIO SUITE 2022. The unit cell has periodic boundary conditions in the *x*- and *y*-axis. Two Floquet ports are employed along the *z*-axis. The LCP and RCP excitations incident vertically to the unit cell.

**Figure 2:**
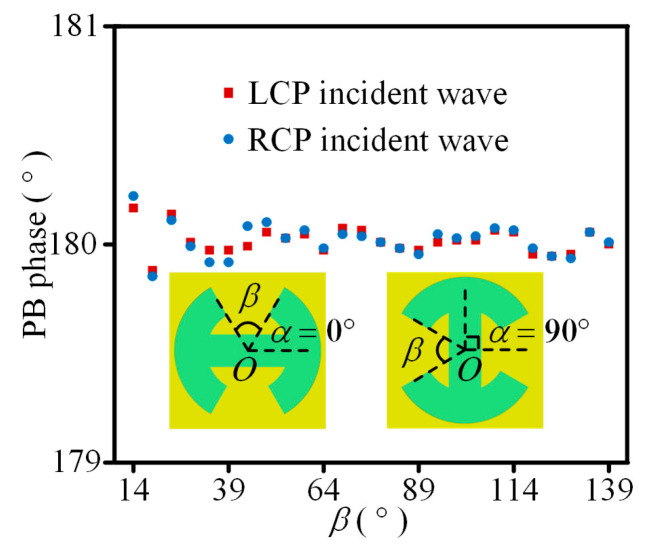
When cross-circularly polarized waves incident vertically to the unit cell, the simulated PB phase of cross-polarized transmitted waves at 23 GHz. *α* = 0° and 90° and *β* linearly varies from 14° to 139° with a step of 5°.

First, to prove that the same PB phase responses can be acquired under cross-circularly polarized waves, *α* is set to 0° and 90°, and *β* linearly varying from 14° to 139° with a step of 5° is taken as an example. When CP wave incidents vertically to the unit cell, Figure 2 shows the simulated PB phase response with various *β* at 23 GHz. It can be seen that phase responses of the cross-polarized transmitted waves are almost the same, and about 180° abrupt phases are obtained when *α* changes from 0° to 90°. Therefore, the same 180° PB phase is achieved by the proposed unit cell under cross-circularly polarized waves.

**Figure 3:**
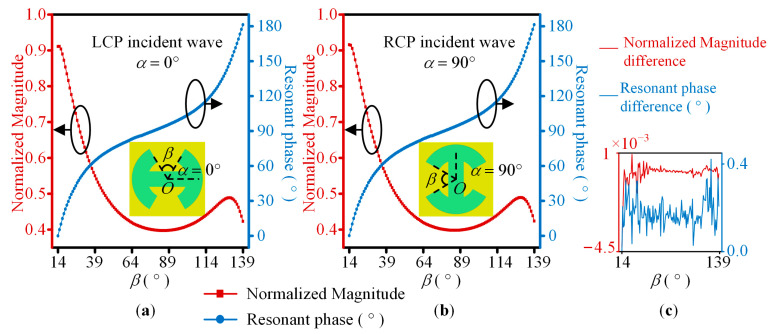
The simulated normalized amplitude and resonant phase of cross-circularly polarized transmitted waves at 23 GHz. (**a**) The LCP incident wave and *α* = 0°; (**b**) the RCP incident wave and *α* = 90°. *β* linearly varies from 14° to 139° with a step of 1°; (**c**) the normalized amplitude and resonant phase differences.

Then, to prove that the same resonant phase responses can be realized under cross-circularly polarized waves, *β* linearly varies from 14° to 139° with a step of 1°. For the LCP and RCP waves, *α* = 0° and 90°are taken as examples, respectively. When CP waves incident vertically to the unit cell, the simulated normalized magnitude and resonant phase of the cross-polarized transmitted wave at 23 GHz are shown in Figure 3a,b. The differences between the LCP and RCP waves are shown in Figure 3c. It can be seen that the simulation results are almost the same. The normalized amplitude is not less than 0.4, and the phase shift can vary between 0° and 180° with *β* changing from 14° to 139°. Therefore, the same resonant phase is realized by the proposed unit cell and the phase shift can cover 0° to 180°.

**Figure 4:**
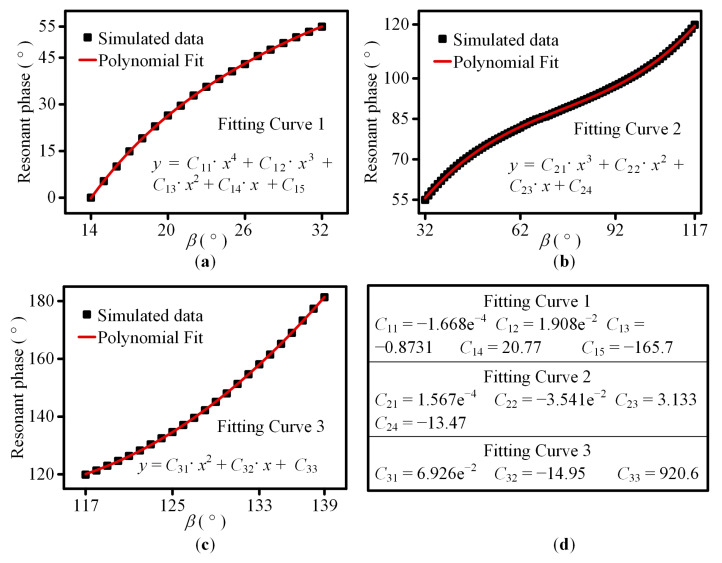
Polynomial fitting curves and coefficients. (**a**) The resonant phase is 0~55° and the corresponding *β* is 14~32°. (**b**) The resonant phase is 55~120° and the corresponding *β* is 32~117°. (**c**) The resonant phase is 120~180° and the corresponding *β* is 117~139°. (**d**) Coefficients of the three polynomial fitting curves. The variables *x* and *y* represent *β* and the resonant phase respectively. *C*_11_, *C*_12_, *C*_13_, *C*_14_, and *C*_15_ are the coefficients of the fitting curve 1. *C*_21_, *C*_22_, *C*_23_, and *C*_24_ are the coefficients of the fitting curve 2. *C*_31_, *C*_32_, and *C*_33_ are the coefficients of the fitting curve 3.

Next, to make the design method more flexible, the fitting relation between the resonant phase and *β* is attained by the polynomial fitting curve method in MATLAB R2017a. According to Figure 3, resonant phase responses are almost the same for the different polarization states of the incident wave and *α*. Therefore, in this paper, the fitting curve is calculated based on the resonant phase curve in Figure 3a. Because a simple polynomial is incapable of achieving good fitting, according to the slope change in the curve, the 0~180° resonant phase is divided into 0~55°, 55~120°, and 120~180° three sections. The corresponding *β* ranges are 14~32°, 32~117°, and 117~139°, respectively. Figure 4 shows the three polynomial fitting curves and the corresponding coefficient. According to Figure 4a–c, the simulation data and the polynomial curve fit well. The corresponding *β* can be easily calculated by the three polynomial fitting curves.

Based on the above results, for cross-circularly polarized incident waves, the same PB and resonant phase responses can be achieved. The abrupt phase introduced by the unit cell can cover 360° by combining PB and resonant phases. According to the required resonant phase, the corresponding unit cell parameter can be easily calculated by the polynomial fitting curves shown in Figure 4. As the arbitrary LP wave can be divided into LCP and RCP waves, the proposed unit cell is polarization insensitive.

### 3.2. Metalens

**Figure 5:**
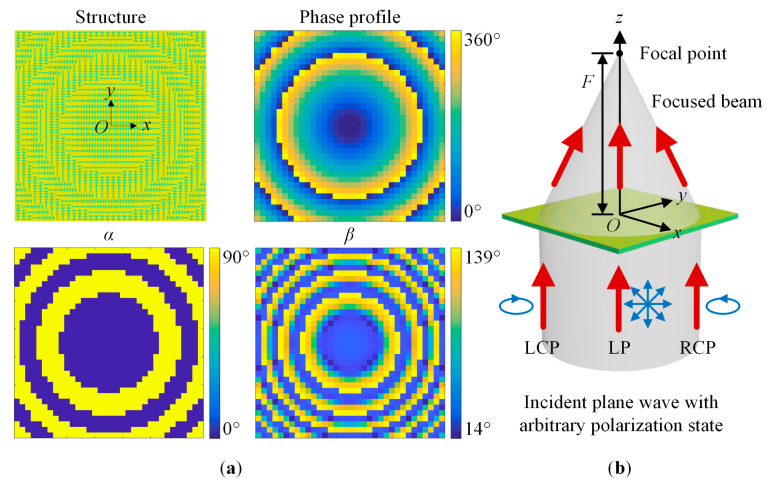
The schematic diagram of the polarization-insensitive metalens. (**a**) The structure, abrupt phase profile, and *α* and *β* distributions. (**b**) Working principle. *α* and *β* represent the rotation angle and the open angle of the SRR slot respectively. *F* is the focal length of the metalens. The red arrows represent the incident wave and the focused wave.

A polarization-insensitive transmissive microwave metalens operating at 23 GHz is devised for verification. The metalens is composed of 34 × 34 unit cells and the focal length *F* is 50 mm. According to the design principle, the required abrupt phase Φ(*x_i_*, *y_i_*), the rotation angle *α*, and the parameter *β* at different positions can be obtained. Figure 5 shows the structure, abrupt phase profile, *α* and *β* distributions, and working principle schematic diagram of the designed metalens. Figure 5b shows that the incident plane wave with an arbitrary polarization state would converge at the focal point.

**Figure 6:**
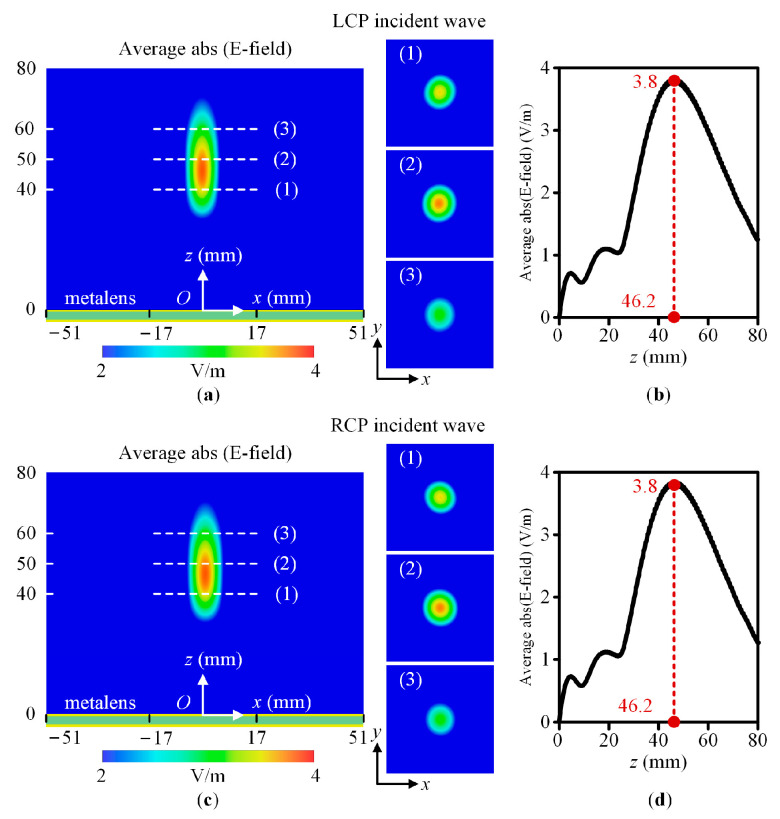
The average absolute value of the electric field amplitude in the near fieldin the XOZ plane with *x* = −51 mm to 51 mm and *z* = 0 mm to 80 mm, the XY planes with *x* = −17 mm to 17 mm, *y* = −17 mm to 17 mm, and *z* = 40 mm, 50 mm, and 60 mm, respectively, and the centerline with *x* = 0 mm, *y* = 0 mm, and *z* = 0 mm to 80 mm. (**a**,**b**) The LCP wave. (**c**,**d**) The RCP wave. Average abs (E-field) represents the average absolute value of the electric field amplitude. The numbers 1, 2, and 3 represent three XY planes.

**Figure 7:**
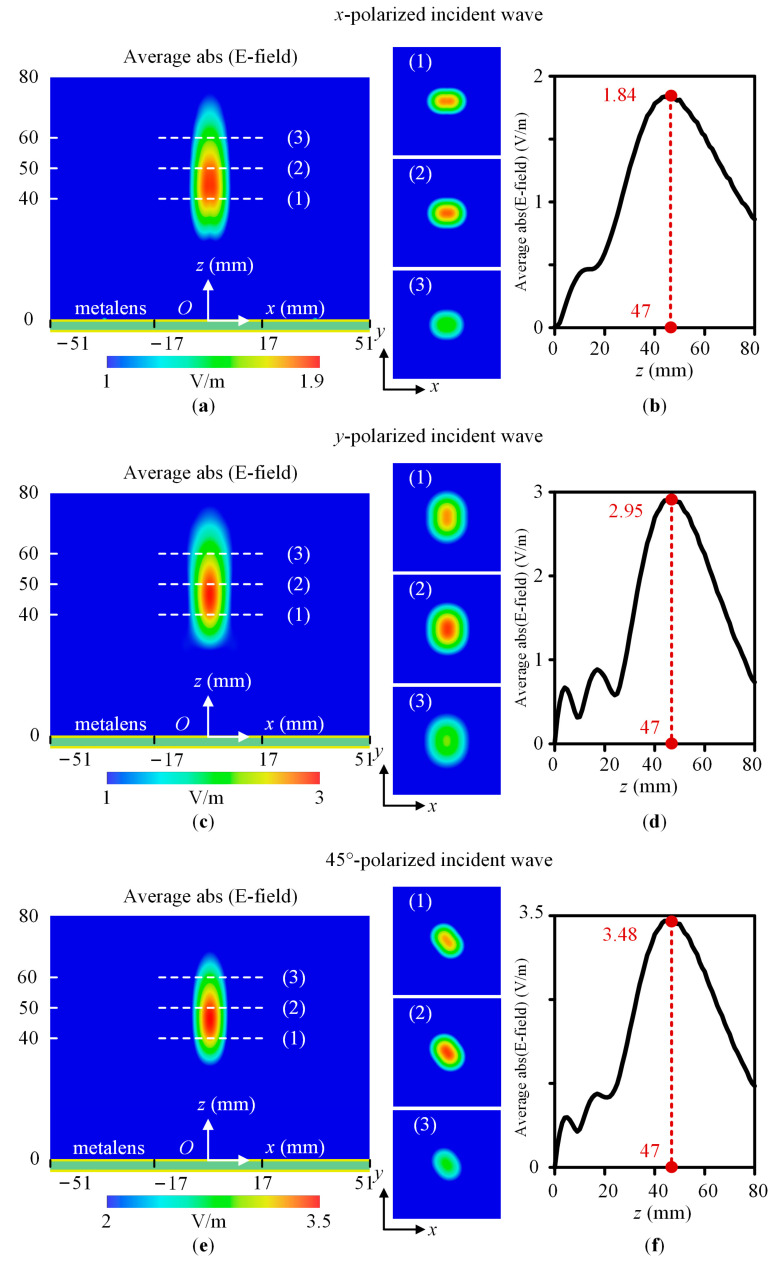
The average absolute value of the electric field amplitude in the near field in the XOZ plane with *x* = −51 mm to 51 mm and *z* = 0 mm to 80 mm, the XY planes with *x* = −17 mm to 17 mm, *y* = −17 mm to 17 mm, and *z* = 40 mm, 50 mm, and 70 mm, respectively, and the centerline with *x* = 0 mm, *y* = 0 mm, and *z* = 0 mm to 80 mm. (**a**,**b**) The *x*-polarized wave. (**c**,**d**) The *y*-polarized wave. (**e**,**f**) The 45°-polarized wave. Average abs (E-field) represents the average absolute value of the electric field amplitude. The numbers 1, 2, and 3 represent three XY planes.

To show the polarization insensitivity achieved by the designed metalens, without loss of generality, we chose incident waves having LCP, RCP, *x*-polarized, *y*-polarized, and 45°-polarized with *f* = 23 GHz. Figure 6 and Figure 7 show the average absolute value of the electric field amplitude in the near field for the CP and LP incident waves, respectively. Figure 6a,c and Figure 7a,c,e show the amplitude distribution of the XY plane with *x* = −51 mm to 51 mm and *z* = 0 mm to 80 mm, and XY planes with *x* = −17 mm to 17 mm, *y* = −17 mm to 17 mm, and *z* = 40 mm, 50 mm, and 60 mm, respectively. Figure 6b,d and Figure 7b,d,f show the amplitude distribution of the metalens centerline with *x* = 0 mm, *y* = 0 mm, and *z* = 0 mm to 80 mm. According to simulation results, the LCP, RCP, *x*-polarized, *y*-polarized, and 45°-polarized normal incident waves are focused around the focal point. The focal lengths are 46.2 mm, 46.2 mm, 47 mm, 47 mm, and 47 mm, respectively. The maximum deviation is 7.6% to the designed focal length *F* = 50 mm. Thus, the designed metalens working at 23 GHz is polarization insensitive.

### 3.3. Orbital Angular Momentum (OAM) Multiplexing Metasurface

**Figure 8:**
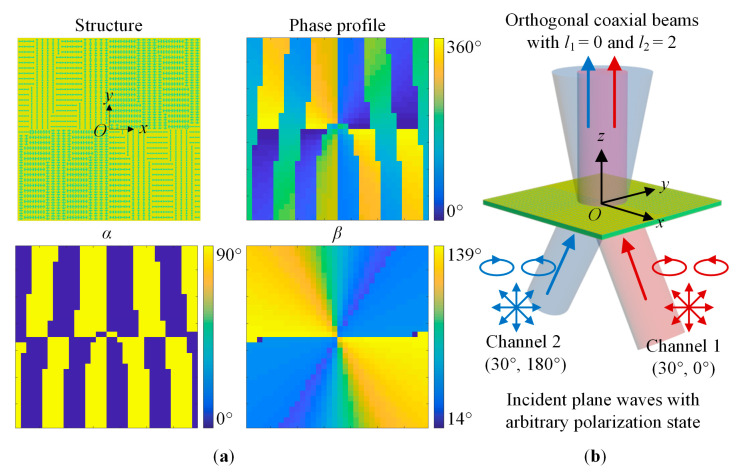
The schematic diagram of the angle-multiplexed metasurface for OAM multiplexing. (**a**) The structure, abrupt phase profile, and *α* and *β* distributions. (**b**) Working principle. The blue and red arrows represent the incident waves of the channels 1 and 2 respectively.

The OAM multiplexing metasurface is composed of 34 × 34 unit cells. *M* = 2, (*θ*_1_, *φ*_1_) = (30°, 0°), (*θ*_2_, *φ*_2_) = (30°, 180°), *l*_1_ = 0, and *l*_2_ = 2. According to the design principle, the required abrupt phase Φ(*x_i_*, *y_i_*), the rotation angle *α* and the parameter *β* at different positions can be obtained. Figure 8 shows the structure, abrupt phase profile, *α* and *β* distributions, and working principle schematic diagrams of the designed OAM multiplexing metasurface. As shown in Figure 8b, when the *m*-channel plane wave with arbitrary polarization state incidents obliquely to the polarization-insensitive multiplexing metasurface at the angle (*θ_m_*, *φ_m_*), in the direction perpendicular to the metasurface, an OAM beam with *l*_m_ would be generated. As OAM beams with different *l* are orthogonal to each other, *M*-channel orthogonal coaxial beams would be realized, that is *M*-channel multiplexing.

**Figure 9:**
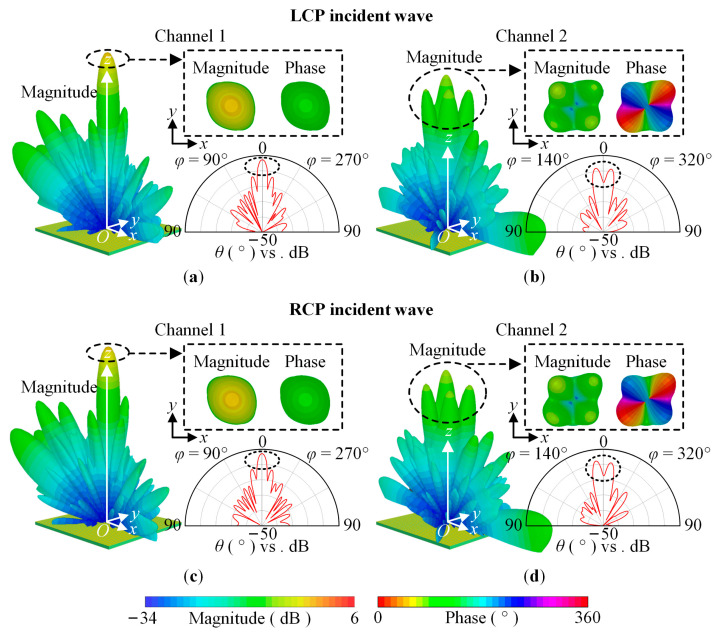
The magnitude and phase of the far field RCS. The cross-polarized transmitted wave for the LCP incident wave: (**a**) Channel 1; (**b**) Channel 2. The cross-polarized transmitted wave for the RCP incident wave: (**c**) Channel 1; (**d**) Channel 2.

**Figure 10:**
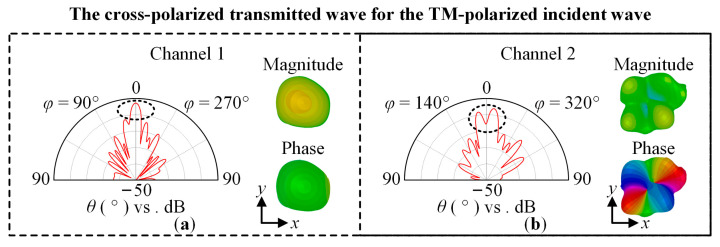

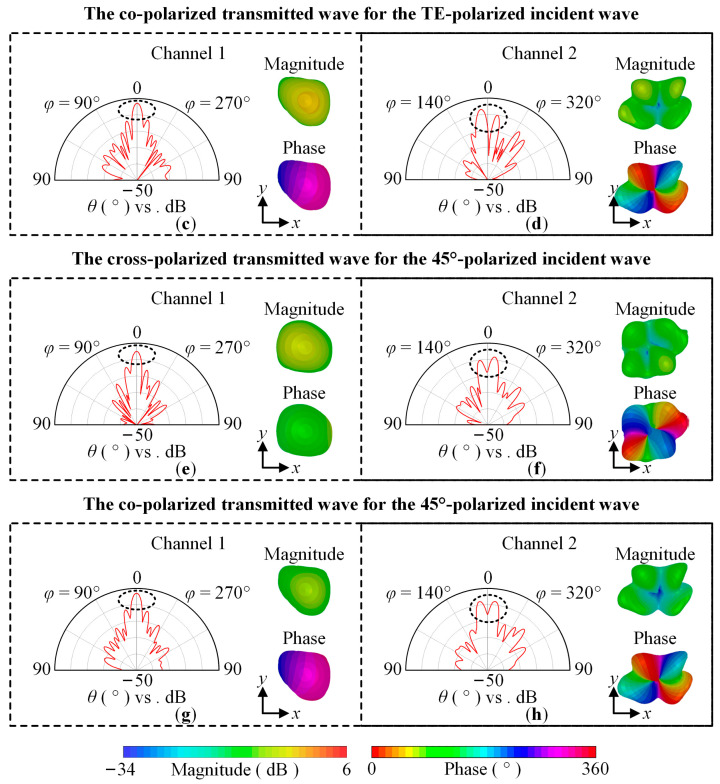
The magnitude and phase of the far field RCS. The cross-polarized transmitted wave for the TM-polarized incident wave: (**a**) Channel 1; (**b**) Channel 2. The co-polarized transmitted wave for the TE-polarized incident wave: (**c**) Channel 1; (**d**) Channel 2. The cross-polarized and co-polarized transmitted waves for the 45°-polarized incident wave: (**e**,**g**) Channel 1; (**f**,**h**) Channel 2.

**Figure 11:**
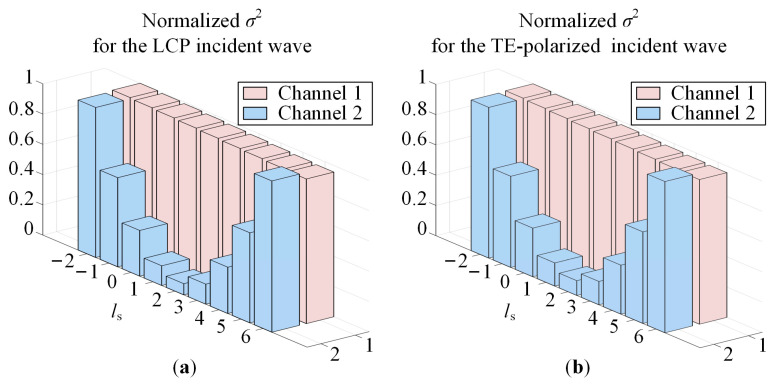
The normalized *σ*^2^. (**a**) LCP incident wave. (**b**) TE-polarized incident wave.

To show the polarization insensitivity achieved by the designed OAM multiplexing metasurface, without loss of generality, we chose incident waves having LCP, RCP, TM-polarized, TE-polarized, and 45°-polarized with *f* = 23 GHz. Figure 9 shows the far-field magnitude and phase of the cross-polarized transmitted wave for the CP incident wave. Figure 10 shows the far-field magnitude and phase of the cross-polarized or co-polarized transmitted wave for the TM-polarized and TE-polarized incident wave, respectively, and the cross-polarized and co-polarized transmitted wave for the 45°-polarized incident wave. The magnitude and phase have the same scaling of −34 dB to 6 dB and 0° to 360°, respectively. Figure 11 shows the OAM purity of the generated beam for the LCP and TE-polarized waves, *θ*_s_ is 5°, *φ*_s_ linearly varies from 0° to 360° with a step of 1°, and *l*_s_ linearly varies from −2 to +6 with a step of +1.

For Channel 1, the three-dimensional and two-dimensional far-field magnitude and phase distribution are shown in Figure 9a,c and Figure 10a,c,e,g. For Channel 2, the simulation results are shown in Figure 9b,d and Figure 10b,d,f,h. According to the simulation results, for the LCP, RCP, TM-polarized, TE-polarized, and 45°-polarized oblique incident waves, in the direction perpendicular to the metasurface, two-channel coaxial beams are generated. For Channel 1, the magnitude is solid and the phase is unchanged. Therefore, the topological charge of the generated beam is 0. For Channel 2, the magnitude is hollow and the phase changes 4π counterclockwise. Therefore, the topological charge of the generated beam is 2. The LCP and TE-polarized waves are taken as examples, the topological charge can also be judged according to Figure 11. For Channel 1, the normalized *σ*^2^ is approximately 1 with different *l*_s_, while for Channel 2, the normalized *σ*^2^ is the smallest with *l*_s_ = +2.

Based on the above results, it can be seen that the designed metalens and OAM multiplexing metasurface working at 23 GHz are polarization insensitive.

## 4. Experimental Demonstration

**Figure 12:**
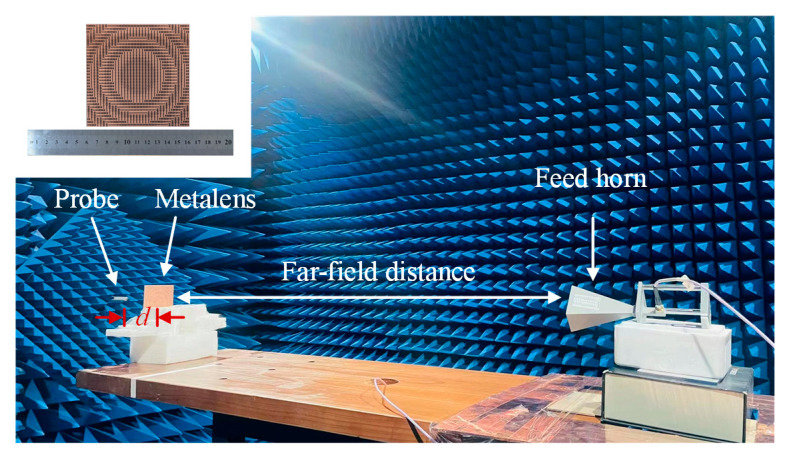
The measurement platform setup.

At last, the polarization-insensitive metalens prototype was fabricated by the printed circuit board (PCB) technology for experimental verification. A 2.93 mm-thick F_4_B265 substrate was adopted over which 0.035 mm-thick copper unit cells were etched. The near-field characteristics were performed in an anechoic chamber. Figure 12 shows the measurement platform. The metalens prototype is set at the far-field position of the feed horn. Therefore, for the metalens, the field generated by the horn is approximately a 23 GHz plane wave. The distance between the probe and the prototype is *d*.

**Figure 13:**
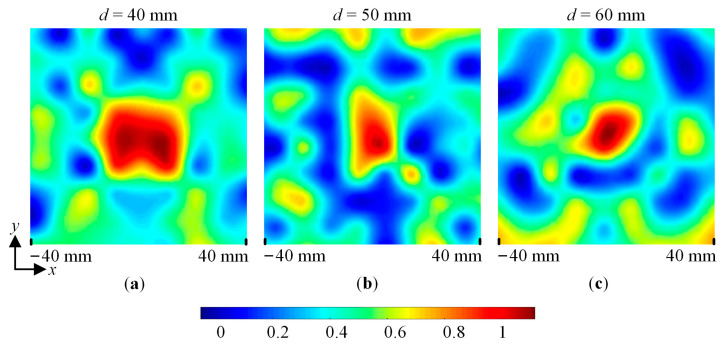
The measured normalized near-field amplitude value of the XY plane with *x* = −40 mm to 40 mm for the *y*-polarized incident wave. (**a**) *d* = 40 mm. (**b**) *d* = 50 mm. (**c**) *d* = 60 mm.

**Figure 14:**
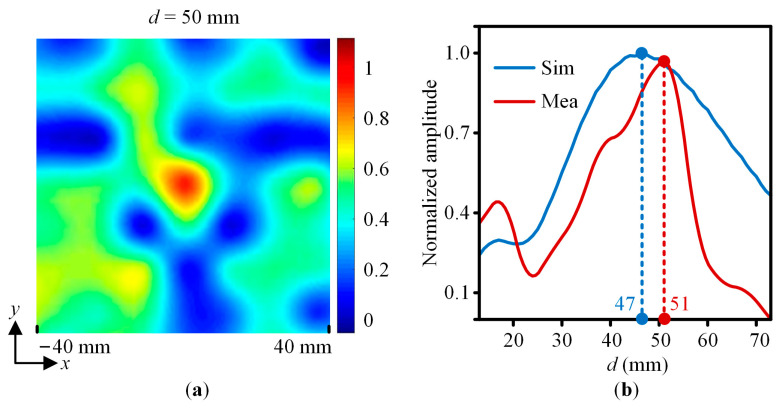
The simulated and measured normalized near-field amplitude value for the 45°-polarized incident wave. (**a**) The XY plane with *x* = −40 mm to 40 mm and *d* = 50 mm. (**b**) The centerline of the metalens with *d* = 13 mm to 73 mm.

For the *y*-polarized incident wave, the measured normalized near-field amplitude distribution of the XY plane with *x* = −40 mm to 40 mm and *d* = 40 mm, 50 mm, and 60 mm are shown in Figure 13. For the 45°-polarized incident wave, Figure 14a shows the measured normalized near-field amplitude distribution of the XY plane with *x* = −40 mm to 40 mm and *d* = 50 mm. Figure 14b shows the simulated and measured normalized near-field amplitude distributions of the metalens centerline with *d* = 13 mm to 73 mm. It is thus clear that the *y*-polarized and 45°-polarized waves converge around the designed focal length *F* = 50 mm. Therefore, the designed metalens working at 23 GHz is polarization insensitive.

## 5. Conclusions

In this paper, a design method for achieving a microwave transmission metasurface with polarization insensitivity is proposed. First, a unit cell is designed, and for LCP and RCP incident waves, the same abrupt phase combining PB and resonant phases can be obtained. As the arbitrarily polarized wave can be decomposed into the LCP and RCP waves, for the metasurface composed of the unit cell, the same phase profile can be realized. Therefore, the metasurface is insensitive to any polarization states at working frequency. For verification, the metalens and OAM multiplexing metasurface are designed. According to the fitting curve, the parameter of the unit cell with the required resonant phase can be easily obtained without redesigning the unit cell which greatly simplifies the design process. Simulation and measurement results verify the designed metasurface is effective for various polarization states at 23 GHz. The proposed method can guide the realization of the polarization-insensitive microwave metasurface with the required functions. The designed polarization-insensitive metalens and OAM multiplexing metasurface have potential applications in high-gain antenna and high-speed wireless communication.

## Data Availability

The data that support the findings of this study are available from the author upon reasonable request.
